# Genetic markers of cardiac autonomic neuropathy in the Kazakh population

**DOI:** 10.1186/s12872-024-03912-0

**Published:** 2024-05-09

**Authors:** Nazira Bekenova, Ainur Sibagatova, Alisher Aitkaliyev, Tamara Vochshenkova, Balzhan Kassiyeva, Valeriy Benberin

**Affiliations:** Gerontology Center, Medical Centre Hospital of President’s Affairs Administration of the Republic of Kazakhstan, Mangilik El 80, Astana City, 010000 Kazakhstan

**Keywords:** Cardiac autonomic neuropathy, Genetic polymorphisms, Kazakh population, Heart rate variability

## Abstract

**Background:**

Cardiac autonomic neuropathy (CAN) is a complication of diabetes mellitus (DM) that increases the risk of morbidity and mortality by disrupting cardiac innervation. Recent evidence suggests that CAN may manifest even before the onset of DM, with prediabetes and metabolic syndrome potentially serving as precursors. This study aims to identify genetic markers associated with CAN development in the Kazakh population by investigating the SNPs of specific genes.

**Materials and methods:**

A case-control study involved 82 patients with CAN (cases) and 100 patients without CAN (controls). A total of 182 individuals of Kazakh nationality were enrolled from a hospital affiliated with the RSE “Medical Center Hospital of the President’s Affairs Administration of the Republic of Kazakhstan”. 7 SNPs of genes FTO, PPARG, SNCA, XRCC1, FLACC1/CASP8 were studied. Statistical analysis was performed using Chi-square methods, calculation of odds ratios (OR) with 95% confidence intervals (CI), and logistic regression in SPSS 26.0. Results: Among the SNCA gene polymorphisms, rs2737029 was significantly associated with CAN, almost doubling the risk of CAN (OR 2.03(1.09–3.77), *p* = 0.03). However, no statistically significant association with CAN was detected with the rs2736990 of the SNCA gene (OR 1.00 CI (0.63–1.59), *p* = 0.99). rs12149832 of the FTO gene increased the risk of CAN threefold (OR 3.22(1.04–9.95), *p* = 0.04), while rs1801282 of the PPARG gene and rs13016963 of the FLACC1 gene increased the risk twofold (OR 2.56(1.19–5.49), *p* = 0.02) and (OR 2.34(1.00-5.46), *p* = 0.05) respectively. rs1108775 and rs1799782 of the XRCC1 gene were associated with reduced chances of developing CAN both before and after adjustment (OR 0.24, CI (0.09–0.68), *p* = 0.007, and OR 0.43, CI (0.22–0.84), *p* = 0.02, respectively). Conclusion: The study suggests that rs2737029 (SNCA gene), rs12149832 (FTO gene), rs1801282 (PPARG gene), and rs13016963 (FLACC1 gene) may be predisposing factors for CAN development. Additionally, SNPs rs1108775 and rs1799782 (XRCC1 gene) may confer resistance to CAN. Only one polymorphism rs2736990 of the SNCA gene was not associated with CAN.

**Supplementary Information:**

The online version contains supplementary material available at 10.1186/s12872-024-03912-0.

## Background

It’s generally accepted that Cardiac autonomic neuropathy (CAN) is a complication of diabetes mellitus (DM) that disrupts cardiac innervation and increases the risk of morbidity and mortality [1–3]. Some sources describe CAN as an autonomic control disorder of the cardiovascular system specifically associated with DM, after excluding other factors [4, 5]. However, emerging evidence suggests that CAN may manifest even before the onset of DM, with prediabetes and metabolic syndrome being potential precursors [5]. Prediabetes, as classified by the World Health Organization, encompasses impaired fasting glucose levels and impaired glucose tolerance [6, 7]. Moreover, some researchers propose that early-onset CAN is more characteristic of insulin-dependent diabetes [8].

Due to limited research on the disease, prevalence data varies, and analysis is primarily based on systematic reviews and meta-analyses. Some studies indicate that CAN may occur in approximately 90% of individuals with DM [3, 9]. Other sources report prevalence rates ranging from 2 to 91% in type 1 diabetes mellitus (DM1) and 25–75% in type 2 diabetes (DM2) [10, 11].

Mortality rates associated with CAN are also significant, as there is a risk of death even at the asymptomatic stage [12]. Studies have demonstrated that the five-year mortality from CAN ranges from 15 to 60% [3, 13].

Regarding gender differences, the incidence of CAN was higher in women compared to men (2.2% and 1.4% in severe cases, 4.7% and 2.6% in moderate cases, respectively) [14]. However, it is crucial to understand the incidence of CAN by gender, especially in the early stages. Since early-stage CAN is often underdiagnosed, available information mainly focuses on differences in heart rate variability (HRV) between men and women, showing that a decrease in HRV is more frequently observed in women [15]. Decreased HRV is considered an early sign of cardiac dysautonomia [1, 10].

As neuropathy originates in the longest nerve fibres, early manifestations of CAN result from impaired innervation of the parasympathetic nervous system, leading to changes in HRV [12, 16]. While many studies emphasize the primary inhibition of the parasympathetic nervous system [6, 17], the disruption of parasympathetic innervation subsequently leads to sympathetic nervous system dominance. It is worth noting that sympathetic dominance is also influenced by dysglycemia [5] and contributes to insulin resistance and hyperinsulinemia in prediabetes [18]. These changes in the sympathetic and parasympathetic nerve fibres innervating the heart and blood vessels result in reduced HRV [19]. Higher resting heart rate and reduced HRV correlate with the risk of developing components of metabolic syndrome and subsequent cardiovascular mortality [20].

Additionally, the denervation of sympathetic nerve fibres starts from the apex of the ventricles and progresses toward the base. Importantly, the reversibility of CAN disorders decreases as the disease advances [6, 12]. Consequently, early markers for the disease are crucial to identify.

It is known that in the pathogenesis of cardiac autonomic neuropathy, not only hyperglycemia and insulin deficiency play a role but also disruptions in lipid metabolism [21], which subsequently influence the reduction of neural blood flow, leading to a decrease in conductivity in the heart. Atrophy of axons and demyelination of nerve fibres, developed due to reduced neural blood flow, also contribute to decreased conductivity in the heart. In this context, it would be interesting to consider certain polymorphisms that have important functional significance in lipid metabolism, myelination of nerve fibres, and insulin resistance.

Among all genes associated with fat mass and obesity, the FTO gene (FTO alpha-ketoglutarate-dependent dioxygenase) stands out with the strongest correlation. According to GWAS studies, it is practically linked to disruptions in lipid metabolism in almost all populations. According to some data, it is considered a predisposition gene for obesity [22, 23].

Following GWAS studies, most obesity loci have been examined in individuals of European descent [22]. Recent studies conducted in an Asian population among 62,245 East Asian participants found a significant association with the rs12149832 polymorphism of the FTO gene (*p* = 4.8 × 10–22) [24].

Participation in the processes of insulin resistance is due to epigenetic modifications of the FTO gene, in particular DNA methylation, which have an inhibitory effect on the PPARG gene [25].

PPARG improves insulin sensitivity by increasing fatty acid stores in fat cells (decreasing lipotoxicity), enhancing the release of adiponectin from fat cells and gain of production nicotinic acid adenine dinucleotide phosphate due to enzyme CD 38 activation [26].

It is known that mutations in this gene, that is, single-nucleotide substitutions, impair insulin sensitivity. Polymorphism rs1801282 of the PPARG gene leads to the replacement of proline with alanine in codon 12 of exon B (C > G; Pro 12 Ala) and causes conformational changes in the protein, and the presence of a minor allele is associated with a decrease in PPARG activity. The C allele of this polymorphism is associated with increased PPARG transcriptional activity and, consequently, insulin hypersensitivity. Although, it’s important to note that there are conflicting opinions about the connection between the rs1801282 polymorphism and diabetes. Studies of its relationship with gestational diabetes showed that rs1801282 reduced the risk of the disease in 50% of cases in the French population, while the studies conducted in Turkey report that rs1801282 did not affect the prevalence of gestational diabetes. However, in both populations, it was associated with excess weight [27–30].

It is known that there are common mechanisms in the pathogenesis of neurodegenerative diseases and complications of diabetes mellitus since these diseases are characterized by autonomic disorders [31, 32]. Polymorphisms rs2736990 and rs2737029 in the SNCA gene are important in the pathogenesis of Parkinson’s disease [33, 34]. However, it has not been studied in other diseases, particularly in CAN.

The understudied polymorphisms rs1108775 and rs1799782 were of great interest due to their investigation in the Kazakh population and their association with ischemic heart disease [35] and reduced risk of thyroid cancer [36], respectively. This study aims to investigate the Single Nucleotide Polymorphisms (polymorphisms) of specific genes to identify genetic markers associated with CAN development in the Kazakh population.

## Materials and methods

### Patients’ selection

The case-control study included 82 patients with CAN (cases) and 100 patients with no CAN (controls). The statistical software Epi Info was used to calculate the sample size. The estimated frequency in the control group was 30%, with an odds ratio of 2.0, confidence level of 95%, and statistical power of 80%. The selection into the group of cases was carried out continuously among the patients from the therapeutic department of the Medical Centre Hospital of the President’s Affairs Administration of the Republic of Kazakhstan consecutively for the period from September 2017 to August 2019 (with the hospital’s turnover at the beginning of the study (2017) being 25,454 people). The control group was also formed from persons who underwent preventive examinations in the same hospital, and in whom the diagnosis of CAN was excluded.

Criteria for inclusion in the group of cases were an established diagnosis of CAN, age of 18 years and older, and Kazakh nationality. Exclusion criteria were a history of genetic disease, hypothyroidism or hyperthyroidism, rhythmic disturbances in the electrical activity of the heart, LVAD placement within the last 3 months, regular alcohol consumption (more than 80 mg/day), anemia (Hb < 110), cancer, kidney disease, severe CVD, liver, end-stage blood, autoimmune diseases that can affect autonomic nerve fibres such as systemic lupus erythematosus, concomitant degenerative disease (e.g., Parkinson’s disease or multiple system atrophy), drugs that can affect the frequency heart contractions such as beta-blockers, verapamil, diltiazem, amiodarone or nitrates, pregnant and lactating women.

The inclusion criteria for the control group were excluding the diagnosis of CAN, age 18 years and older, and the Kazakh population. Exclusion criteria were like those in the case group.

Out of the entire patient population, 82 individuals exhibited indications of CAN based on the daily Electrocardiogram (ECG) monitoring conducted through Holter monitoring. The Medilog DARWIN ECG Holter monitoring system from Switzerland was utilized to perform 24-hour Holter monitoring. Data collection focused on the following parameters:

- Average Standard Deviation of NN Intervals (SDNN av) (ref. interval 53–279 m/sec).
- Median of Standard Deviation of NN Intervals (SDNN med) (ref. interval > 53.8 m/sec).
- Average Standard Deviation of Average NN Intervals (SDANN av) (ref. interval 45–261 m/sec).
- Average Root Mean Square of Successive Differences (RMSSD av) (ref. interval 7–103 m/sec).
- Median of Root Mean Square of Successive Differences (RMSSD med) (ref. interval 28.8–71.9) m/sec)
- Average Percentage of NN Intervals differing by more than 50 ms (pNN50 av) (ref. interval 0-137%).
- Median Percentage of NN Intervals differing by more than 50 ms (pNN50 med) (ref. interval 6.0-44.1%).
- High-Frequency Power (HF) (> 56.4).
- Low-Frequency Power (LF) (< 43.6).
- The ratio of High-Frequency Power to Low-Frequency Power (HF/LF) (< 0.8).

The above values were used as normal values and were used to select patients for the control group, and deviations from these values were used for the diagnosis of CAN and selection for the case group. CAN was characterized as deviations in three or more Heart Rate Variability (HRV) measurements (such as SDNN, RMSSD, HF, LF, and HF/LF) [37, 38].

HT was determined by observing an elevation in systolic blood pressure (SBP) equal to or greater than 140 mm Hg, and/or diastolic blood pressure (DBP) equal to or greater than 90 mm Hg, either through daily monitoring of blood pressure or by assessing the consistent use of antihypertensive medication. The BTL-08 ABPM ambulatory blood pressure recorder, manufactured in Great Britain, was used for the daily monitoring of blood pressure.

The diagnosis of DM2 was determined based on the clinical protocol established by the Ministry of Health of the Republic of Kazakhstan [39]. Specifically, laboratory parameters including fasting glucose concentration (≥ 6.1 mmol/l) and glycosylated hemoglobin (≥ 6.5% or 48 mmol/mol) were utilized for diagnosis.

BMI was calculated by dividing weight in kilograms by the square of height in meters.

The following anamnestic data were analyzed: Age, Sex, and a habit of smoking. Clinical data were also evaluated (glucose concentration, triglyceride level). Glucose level was determined under fasted conditions from venous blood. Blood samples were collected from the cubital vein in the treatment room after a 12-hour fast. The plasma was separated by spinning at 1000×g (4 C) for 10 min. To conduct further biochemical analysis, the plasma was stored at -30 C. The serum obtained after centrifugation was used for analysis on the same day as the blood collection. The levels of glucose, total cholesterol, TG, HDL, and LDL were measured using the enzymatic method on an Architect’s 8000 automatic biochemical analyzer manufactured by Abbott Laboratories in the USA.

### Isolation of DNA

DNA extraction from blood samples was carried out using kits from the DSMZ-German Collection of Microorganisms and Cell Cultures (Germany, catalogue number ACC11) following the manufacturer’s instructions. DNA extraction was performed automatically using the AutoMate Express™ Instrument. The iPrep™ Purelink™ gDNA Blood Kit was used for this purpose. Initially, tubes were prepared and labelled according to the DNA samples. Subsequently, the Qubit® working solution was created by diluting the Qubit® dsDNA BR Reagent in the Qubit® dsDNA BR Buffer at a ratio of 1:200 for each patient. Then, 2 µl of the buffer and reagent mix were combined with 2 µl of DNA. The concentration of DNA was assessed using the Qubit™ 4 Fluorometer with the Qubit® dsDNA BR Assay Kits.

### Genotyping

Considering that gene polymorphisms in CAN are poorly studied, the selection of candidate genes and their polymorphisms was carried out from the genetic markers involved in the pathogenesis of age-associated diseases: neurodegenerative diseases, atherosclerosis, DM, obesity, arterial hypertension, breast cancer and prostate in the database of GWAS studies.

All study participants were genotyped for 120 polymorphisms that were identified through a genome-wide search. After a preliminary analysis of 120 polymorphisms with cardiac autonomic neuropathy, an association of 7 polymorphisms was shown: rs12149832 of the FTO gene, rs2737029, rs2736990 of the SNCA gene, rs1801282 of the PPARG gene, rs1799782 of the XRCC 1 gene, rs13016963 of the gene FLACC 1, rs1108775 LINCO 1924, which were further analyzed using logistic regression with adjustment for age and sex. Genotyping was carried out using the innovative OpenArray technology, which enables reactions in very small volumes. Specifically designed OpenArray slides, each containing 3,072 data points, were utilized in this process. To perform genotyping, the previously extracted DNA samples were combined with the reaction mixture in a 384-well sample plate. For each sample, 3.0 µl of OppenArray Real-time master mix and 2.0 µl of DNA sample with a concentration of 50 ng/µl were required. The total volume of the reaction mixture per well was 5 µl, and each sample was duplicated. The plate was thoroughly mixed using a shaker and then centrifuged. Probes were designed using the QuantStudio OpenArray AccuFill Plate Configurator and dried assays were provided in the designated throughholes of the genotyping plates. These plates were specifically designed with two allele-specific probes, a minor groove binder, and two PCR primers to ensure accurate and precise genotyping calls. The OpenArray technology utilizes nanoliter fluidics and can be customized with up to 3,072 through-holes in six different formats.

A plate setup file was created to outline the protocol for the applied samples, including analysis information. This protocol was uploaded into the QuantStudio™ 12 K Flex software to generate and conduct the experiment. The prepared chips were inserted into the QuantStudio 12 K Flex using disposable genotyping blocks. Subsequently, the amplification reaction took place using real-time PCR microfluidic technology. The obtained data from the amplification reaction were analyzed using the online tools provided by the Thermo Fisher Cloud service. The results of the bioinformatic analysis allowed for the classification of the studied genes as homozygotes for the major allele, homozygotes for the minor allele, or heterozygotes.

### Statistical analysis

The dataset for the analysis consisted of personal information, laboratory data, and genotyping data from a total of 182 individuals. The analysis was performed using SPSS (IBM) version 26.0. Quantitative data were presented as means, medians, upper and lower quartiles (M + SD), and Me (Q1, Q3) and were used as continuous variables. Qualitative data is presented as frequencies and proportions. Were dichotomized: gender (male/female), smoking (smoker/non-smoker), and presence of diabetes mellitus (yes/no).

Quantitative data with non-normal distribution were analyzed using the non-parametric Mann–Whitney test for independent groups, and the results were reported as median (Q1; Q3). The normality of the data distribution was assessed using the Shapiro-Wilks criterion. Dichotomous and categorical variables were analyzed using the Chi-square test. A significance level of *p* < 0.05 was considered for determining statistically significant differences.

The association of CAN with gene polymorphisms was performed by comparing groups of patients with CAN (case) and without a diagnosis of CAN (control) using binary logistic regression. The association was appreciated using four inheritance models: dominant, co-dominant, recessive, and log-additive models. Adjustments were made for age and gender since there were no significant differences between the case and control groups in terms of the studied parameters. The analysis was performed using SPSS (IBM) version 26.0.

## Results

The mean patients’ age in groups with and without CAN did not differ significantly and amounted to 54.6 ± 9.1 and 54.7 ± 10.6 years old, respectively. There were no differences between the groups in terms of gender, BMI, and the number of smokers and non-smokers. There were no differences in cholesterol levels, low-density lipoprotein (LDL), high-density lipoprotein (HDL), glucose and glycated haemoglobin and triglycerides among the groups. Moreover, there were no significant differences in heart rate, respiratory rate, diastolic blood pressure, and systolic blood pressure. As for the prevalence of CAN in the population of patients with DM2, it occurred in 1/3 of patients (Table 1).

**Table 1:** Clinical and demographic parameters of patients

	Case (*n* = 82)	Control (*n* = 100)	*p*
Age (M + SD)	54.6 ± 9.1	54.7 ± 10.6	0.97^a _^
Gender (absolute value, %)			
male	52 (63.4%)	60 (60%)	0.64^b _^
female	30 (36.6%)	40 (40%)	
Smoking (absolute value, %)			
nonsmokers	70(85.4%)	82 (82%)	0.54^b _^
smokers	12(14.6%)	18	
BMI, kg / m2 Me (Q 1, Q 3)	28, 8 (27.1–30.9)	30.6 (27.0–32.0)	0.13 ^s^
Heart rate (bpm)	72 (68–76)	74 (70–78)	0.44 ^s^
Respiratory rate (per minute)	17 [16–18]	17 [16–18]	0.46 ^s^
SBP (mm Hg)	130 (120–130)	130 (120-131.3)	0.99 ^s^
DBP (mm Hg)	80 (80–90)	80 (80–90)	0.41 ^s^
Blood glucose (mmol/L)	6.5 (5.5–9.4)	6.2 (5.4–8.5)	0.49 ^s^
glycosylated haemoglobin (%)	6.68 (5,81 − 7,61)	6.32 (5,64 − 8,42)	0.66 ^b^
Triglyceride (mmol/L)	1.66 (1.23–2.54)	1.67 (1.22–2.62)	0.92 ^s^
Cholesterol	5,58 (4,39 − 6,17)	5,38 (4,72 − 6,27)	0,95
LDL	3,35 (2,50 − 4,08)	3,34 (2,79 − 3,98)	0,85
HDL	1,12 (0,97 − 1,34)	1,20 (1,01–1,40)	0,14
DM2 presence (abs, %)			
Yes	26 (31.7%)	45 (45%)	0.07^b _^
No	56 (68.3%)	55 (55%)	
DM duration	Case (*n* = 26)	Control (*n* = 45)	p
1–5 years	12 (46,2%)	21 (46,6%)	0,60 ^b^
6–10 years	9 (34.6%)	19 (42.3%)	
More than10 years	5 (19.2%)	5 (11.1%)	

a - Student’s t-test was used for comparisons

b - comparisons were made using the Chi-square test

c - Mann - Whitney U-test was used to compare mean values

M + SD – mean and standard deviation

Me – median; Q 1–25 quartile, Q 3–75% quartile

### Alleles and genotypes distribution of in hardy- weinberg equilibrium

As a result of the study, out of 120 polymorphisms in a preliminary genetic analysis, 7 polymorphisms were associated with cardiovascular autonomic neuropathy: rs2736990, rs12149832, rs1108775, rs2737029, rs1801282, rs1799782, rs 13,016,963. All 7 polymorphisms were in Hardy- Weinberg equilibrium both in the case group and in the control group (*p* > 0.05). These polymorphisms were further analyzed using four models: codominant, dominant, recessive, and additive.

The binary logistic regression analysis was performed under different inheritance models (codominant, dominant, recessive, and log-additive) in addition; adjustments for gender and age were made (Table 2)).

**Table 2:** Relationship between the SNPs and CAN under multiple models of inheritance

SNP ID	Model	Genotype	Case	Control	Crude analysis		Adjusted*	
					OR (95% CI)	p-value	OR (95% CI)	p-value
rs2736990	Codominant	AA.	12 (14.6%)	10(10%)	1.24(0.37–3.30)	0.66	1.34(0.48–3.70)	0.57
		AG.	40 (48.8%)	59(59%)	0.70(0.37–1.33)	0.28	0.68(0.35–1.32)	0.25
		GG	30 (36.6%)	31(31%)	1	0.36	1	0.28
	Dominant	GG	30 (36.6%)	31(31%)	1	0.43	1	0.41
		AA + AG	52(63.4%)	69(69%)	0.78 (0.42–1.44)		0.77(0.41–1.45)	
	Recessive	AA.	12 (14.6%)	10(10%)	1.28(0.69–2.38)	0.43	1.30(0.69–2.45)	0.41
		GG + AG	70(85.4%)	90(90%)	1		1	
	Log-additive	1,2,3	-	-	0.98 (0.62–1.54)	0.92	1.00(0.63–1.59)	0.99
rs12149832	Codominant	GG	6 (7.3%)	14(14%)	1	0. 03	1	0.02
		GA	30 (36.6%)	49(49%)	1.43(0.50–4.12)	0.51	1.51(0.48–4.79)	0.49
		AA.	46 (56.1%)	37(37%)	2.90(1.02–8.29)	0.05	3.22(1.04–9.95)	0.04
	Dominant	GG	6(7.3%)	14 (14%)	1	0.16	1	0.13
		AA-GA	76(92.7%)	86 (86%)	2.06(0.76–5.63)		2.32(0.78–6.92)	
	Recessive	AA.	46(56.1%)	37(37%)	2.18(1.19–3.95)	0.01	2.31(1.26–4.25)	0.007
		GG-GA	36(43.9%)	63(63%)	1		1	
	Log-additive	1,2,3	-	-	1.82(1.15–2.89)	0,01	1.93(1.20–3.10)	0,01
rs1108775	Codominant	AA.	27 (32.9%)	22 (22%)	1	0.01	1	0.02
		AG.	48 (58.5%)	53 (53%)	0.74(0.37–1.46)	0.39	0.73(0.37–1.46)	0.38
		GG	7 (8.5%)	25 (25%)	0.23(0.08–0.63)	0.0 04	0.24(0.09–0.68)	0.007
	Dominant	AA.	27 (32.9%)	22 (22%)	1	0.10	1	0.11
		GG-AG	55(67.1)	88 (88%)	0.58(0.29–1.11)		0.58(0.30–1.13)	
	Recessive	GG	7 (8.5%)	25 (25%)	0.30 (0.11–0.69)	0.005	0.30(0.12–0.75)	0.009
		AA-AG	75 (91.5%)	75 (75%)	1		1	
	Log-additive	1,2,3	-	-	0.5 2(0.33–0.83)	0.01	0.51(0.32–0.82)	0,01
rs2737029	Codominant	CC	40 (48.8%)	33 (33%)	0.57(0.24–1.31)	0.55	0.55(0.23–1.31)	0.52
		TC	27 (32.9%)	51 (51%)	1.29(0.56-3.0)	0.19	1.32(0.56–3.18)	0.18
		TT	15 (18.3%)	16 (16%)	1	0.43	1	0.35
	Dominant	TT	15 (18.3%)	16 (16%)	1	0.68	1	0.68
		CC- TC	67 (81.7%)	84 (84%)	0.85(0.39–1.85)		0.84(0.38–1.87)	
	Recessive	CC	40 (48.8%)	33 (33%)	1.93(1.06–3.53)	0.03	2.03(1.09–3.77)	0.03
		TT- TC	42 (51.2%)	67 (67%)	1		1	
	Log-additive	1,2,3	-	-	1.30(0.86–1.96)	0.21	1.29(0.85–1.95)	0,23
rs1801282	Codominant	CC	54 (65.9%)	80 (80%)	1	0 0.09	1	0.06
		CG	26 (31.7%)	18 (18%)	2.14(1.07–4.28)	0.03	2.56(1.19–5.49)	0.02
		GG	2 (2.4%)	2 (2%)	1.48(0.20-10.84)	0.70	1.52(0.20-11.47)	0.69
	Dominant	CC	54 (65.9%)	80 (80%)	1	0.03	1	0.02
		GG-CG	28 (34.1%)	20 (20%)	2.07 (1.06–4.05)		2.45(1.17–5.12)	
	Recessive	CC-CG	80(97.6%)	98(98%)	1	0.84	1	0.93
		GG	2(2.4%%)	2(2%)	1.23(0.17–8.89)		1.09(0.15–8.08)	
	Log-additive	1,2,3	-	-	1.81(0.99–3.30)	0,06	1.85(0.98–3.50)	0,07
rs1799782	Codominant	AA.	1 (1.2%)	4 (4%)	0.23(0.03–2.12)	0.20	0.26(0.03–2.41)	0.23
		AG.	17 (20.7%)	37 (37%)	0.42(0.22–0.83)	0.01	0.43(0.22–0.84)	0.02
		GG	64 (78.1%)	59 (59%)	1	0.02	1	0.03
	Dominant	GG	64 (78.1%)	59 (59%)	1	0.007	1	0.009
		AA -AG	18(25%)	41(41%)	0.41(0.21–0.78)		0.41(0.21–0.80)	
	Recessive	AA.	1 (1.2%)	4 (4%)	0.30(0.03–2.70)	0.28	0.34(0.04–3.12)	0.34
		GG -AG	81(98.8%)	96(96%)	1		1	
	Log-additive	1,2,3	-	-	0.44(0.24–0.80)	0.01	0.45(0.24–0.82)	0.01
rs13016963	Codominant	AA.	18 (22%)	15 (15%)	2.22(0.96–3.66)	0.06	2.34(1.00-5.46)	0.05
		AG.	38 (46.3%)	37 (37%)	1.89(0.98–3.66)	0.06	1.97(0.98–3.94)	0.06
		GG	26 (31.7%)	48 (48%)	1	0.08	1	0.07
	Dominant	GG	26 (31.7%)	48 (48%)	1	0.03	1	0.02
		AA-AG	56 (68.3%)	52(52%)	1.98(1.08–3.65)		2.09(1.11–3.94)	
	Recessive	AA.	18 (22%)	15 (15%)	1.59 (0.75–3.40)	0.23	1.68(0.78–3.63)	0.18
		GG-AG	64 (78%)	85 (85%)	1		1	
	Log-additive	1,2,3	-	-	1.55(1.03–2.32)	0.04	1.56(1.04–2.36)	0.03

*- Correction for gender, age, and BMI

### Association rs12149832 and FTO gene (FTO alpha-ketoglutarate dependent dioxygenase) with CAN

During the study, it was found that the AA genotype of the rs12149832 FTO gene polymorphism nearly triples the risk of developing CAN in the codominant model (*p* = 0.05). Furthermore, after adjusting for sex and age, BMI the risk increases to 3.2 times (*p* = 0.04). When analyzed in the recessive model, the risk of developing CAN in carriers of the AA genotype was also increased, but to a lesser extent than in the codominant model, both before correction and after correction (2.18(1.19–3.95), *p* = 0.01) and (2.31(1.26–4.25), *p* = 0.007), respectively. The results from the additive model also suggest that this polymorphism may contribute to the predisposition to CAN (Table 2).

### rs1801282 association with PPARG gene (peroxisome proliferator-activated receptor gamma) with CAN

According to the results of our study shown in Table 2, rs1801282 of the PPARG gene was also associated with CAN. In the codominant model, the heterozygous CG variant more than doubled the risk of developing CAN (*p* = 0.03). In the dominant model, CG and GG genotypes also increased the risk of developing CAN (*p* = 0.03). After adjusting for age and sex, BMI the OR indicators in the codominant and dominant models did not change, and it was also found that CG and GG genotypes increase the risk of developing CAN in the respective models (*p* = 0.02).

### Association of the SNCA gene (synuclein alfa gene) SNP with CAN

Of the two SNCA gene polymorphisms, only rs2737029 was associated with CAN (the results are demonstrated in Table 2). According to the results, the CC genotype increases the risk of development of CAN two times (1.93 (1.06–3.53), *p* = 0.03). After adjusting for age and gender, BMI the scores did not change significantly. However, in other models, no statistically significant association of CAN with rs2737029 of the SNCA gene was found.

The study of the rs2736990 polymorphism did not reveal a statistically significant association with CAN in any of the models.

### Association of rs1108775 with CAN in patients

In codominant and recessive models, the GG genotype of the rs1108775 acted as a protective factor and reduced the risk of developing CAN (0.24(0.09–0.68), *p* = 0.007 and 0.30(0.12–0.75), *p* = 0.009, respectively) shown in Table 2. However, no statistically significant association of CAN with rs1108775 was found in dominant models. The additive model showed that this SNP reduces the chances of developing CAN (0.51 (0.32–0.82), *p* = 0.01 after correction).

### The XRCC 1 gene (X-ray repair cross-complementing 1) with CAN

In the codominant model, the study of the rs1799782 polymorphism of the XRCC 1 gene showed that the presence of the AG genotype reduced the risk of developing CAN (0.43 (0.22–0.84), *p* = 0.02) after correction. In the dominant model, it was found that carriers of the AA and AG genotypes had low chances of developing CAN (0.41 (0.21–0.80), *p* = 0.009). The results of the additive model also show that this polymorphism may reduce the chances of developing CAN (0.45 (0.24–0.82), *p* = 0.01) (Table 2).

### Association of rs13016963 of the FLACC 1 (flagellum associated containing coiled-coil domains 1) gene with CAN

In the codominant model, the rs13016963 polymorphism was not associated with cardiac autonomic neuropathy. However, after correction for gender, age, and BMI, a statistically significant association was observed, with the AA genotype increasing the risk of developing CAN by more than 2 times (2.34 (1.00-5.46), *p* = 0.05).

The rs13016963 of the FLACC 1 gene showed that the AA and AG genotypes in the dominant model increase the risk of developing CAN almost two times (2.06 (1.09–3.85), *p* = 0.03) according to the results in Table 2. The additive model shows that with an increase in the number of risk alleles by one, the chances increase by 1.5 times (1.56 (1.04–2.36) *p* = 0.032).

## Discussion

Polymorphisms rs2737029 of the SNCA Gene, rs12149832 of the FTO Gene, rs1801282 of the PPARG GENE, rs13016963 of FLACC1 can be predisposition factors to the development of CAN, and resistance factors can be polymorphisms rs1108775 and rs1799782 of XRCC 1 gene.

According to our data, the rs2737029 polymorphism of the SNCA gene was associated with CAN. There are only a small number of studies in the literature on the rs2737029 of the SNCA gene, according to which it is associated with a susceptibility to Parkinson’s disease [33]. There is some evidence that HRV is altered when dopamine signalling is deficient in the brain [40]. In addition, animal studies have shown that electrical stimulation of the substantia nigra increases blood pressure and heart rate in awake and anesthetized animals. The important role of dopamine in the work of the heart is also evidenced by the receptors for serotonin and dopamine found on myocardial cells and the walls of blood vessels, on the terminals of cholinergic, noradrenergic, and other nerve fibres, in the nerve centres that regulate the work of the heart and other visceral functions. However, this is not a statement that the rs2737029 affects the decrease or increase in dopamine in cells. All these data motivate further research aimed at studying the rs2737029 polymorphism of the SNCA gene in the pathogenesis of CAN, particularly HRV about dopamine receptors.

Moreover, according to the results of our study, the AA genotype of rs12149832 of the FTO gene is associated with a predisposition to CAN. Unfortunately, there are no studies aimed at studying the rs12149832 polymorphism of the FTO gene in CAN, but there is evidence that the AG genotype of rs12149832 predisposes to obesity [24]. However, there is evidence that an elevated BMI is closely associated with CAN [41]. CAN reduces the ability of the autonomic nervous system to provide effective communication between the peripheral (including the gastrointestinal tract) and the central nervous system, which may contribute to further weight gain, obesity, and deterioration of autonomic function by reducing postprandial neurohumoral stimuli that impair satiety [42]. However, in our study, patients with CAN were not identified as obese based on mean BMI values. Though, as it was mentioned this gene is involved in the processes of insulin sensitivity in conjunction with the PPARG gene.

In our study, the CG genotype of the rs1801282 polymorphism of the PPARG gene increases the risk of developing CAN. Although to date there are no studies aimed at studying this polymorphism with CAN, there is evidence that the C allele of this polymorphism increases insulin sensitivity by increasing the transcriptional activity of PPARG, while the presence of the minor allele has the opposite effect [27].

It is believed that HRV is more related to insulin sensitivity than to the presence of diabetes [43]. This is particularly true in Asian populations, who tend to have low body weight and reduced insulin sensitivity compared to European populations. Possibly, in patients with cardiac neuropathy in our sample, there is dysfunction in insulin sensitivity, with a tendency towards a decrease of insulin sensitivity. Conducting further research may allow us to elucidate the association of this gene with cardiac neuropathy through insulin sensitivity. It should be noted that the presence of diabetes in only 1/3 of the patients may indicate that the reduction in heart rate variability in our sample was not always associated with diabetes.

We found that the rs1108775 in our population reduced the risk of developing CAN. At the same time, according to some authors, the rs1108775 polymorphism was not associated with the risk of developing coronary heart disease in the Kazakh population.

Furthermore, in our investigation, we observed a significant association between two polymorphisms, rs1799782 and rs13016963, and the CAN signs. Previous research has identified these polymorphisms as factors contributing to the development of various types of cancer. Specifically, rs1799782 has been linked to breast [44], colorectal [45], thyroid [36], and lung cancer, and has been shown to impact cancer treatment and overall survival rates [46]. In addition, there is a study that demonstrates a general association of this polymorphism with a combination of various oncological diseases. Arg194Trp (C > T, rs1799782) variant is associated with altered DNA repair activity [47]. Such polymorphisms resulting in amino acid changes can potentially impact the efficiency of DNA repair and hold functional significance. Although the precise functional consequences of these polymorphisms remain unclear, certain studies suggest that amino acid substitutions within evolutionarily conserved regions can influence protein capabilities [48].

rs13016963 has also been implicated in several studies as a genetic factor associated with oral squamous cell carcinoma [49], melanoma [50], esophageal squamous cell carcinoma [51], and lung cancer [52]. Genetic variations that affect the expression or function of caspase-8 may potentially influence tumorigenesis through these mechanisms [53].

At this stage of knowledge, comprehending how rs1799782 and rs13016963 are associated with CAN is challenging. However, it is known that the development of DM2 and oncogenesis are interconnected processes. Publications exist that discuss shared genetic, phenotypic, and environmental factors between DM2 and cancer s [54, 55]. Furthermore, in the pursuit of new targets for DM treatment, articles have been published identifying CASP8 as a potential therapeutic target [56]. Additionally, there is evidence linking XRCC1 to an association with DM2 through its role in DNA repair [57]. Hence, the XRCC1 and CASP8 genes are involved in fundamental cellular processes, namely DNA repair and apoptosis. Consequently, there is evidence supporting the involvement of these proteins in the processes accompanying DM2, which could potentially explain their association with CAN.

The limitations of our study include a small sample size, which makes it possible to identify only relatively strong relationships. However, even this sample size allowed us to identify statistically significant differences. It should also be noted that the recruitment of patients was carried out only within the same medical organization. Because of this, we cannot extrapolate our results to the entire population. The third drawback can be attributed to the fact that different SNPs of different genes that are not related to each other have been studied. The study of several polymorphisms of one gene, located close to each other, and their influence on protein production makes it possible to identify certain patterns in the pathogenesis of the disease.

Although our study may have certain limitations, it also possesses notable strengths. For the first time, the genetic markers of CAN in Kazakhs have been studied and identified as factors of predisposition to CAN based on gene polymorphisms.

Thus, the genetic markers identified in our study may have functional significance in the pathogenesis of cardiac autonomic neuropathy. These patterns are likely to be explained by individual insulin sensitivity and impaired conductivity in nerve endings. All of this needs the conduction of further research in this direction, which could contribute to the prevention of the disease through individually tailored therapy.

### Electronic supplementary material

Below is the link to the electronic supplementary material.

Supplementary Material 1

## Data Availability

Underlying data OSF: Genetic Markers of cardiac autonomic neuropathy in the Kazakh population. Identifier: DOI 10.17605/OSF.IO/Y3U85 This project contains the following underlying data: https://osf.io/y3u85/ Data file 1. Database of Dependent and Independent Variables.xlsx. Data are available under the terms of the Creative Commons Zero “No rights reserved” data waiver (CC0 1.0 Public domain dedication). Software: The presented analysis was performed using SPSS (IBM) version 26.0. the software is available from: https://www.ibm.com/products/spss-statistics.

## Abbreviations

CAN: Cardiac autonomic neuropathy
SNP: Single Nucleotide Polymorphisms
Polymorphisms: Single Nucleotide Polymorphisms
DM: Diabetes mellitus
DM1: Type 1 diabetes mellitus
DM2: Type 2 diabetes
HRV: Heart rate variability
ECG: Electrocardiogram
SDNN av: Average Standard Deviation of NN Intervals
SDNN med: Median of Standard Deviation of NN Intervals
SDANN av: Average Standard Deviation of Average NN Intervals
RMSSD av: Average Root Mean Square of Successive Differences
RMSSD med: Median of Root Mean Square of Successive Differences
pNN50 av: Average Percentage of NN Intervals differing by more than 50 ms
pNN50 med: Median Percentage of NN Intervals differing by more than 50 ms
HRV: Heart Rate Variability Triangular Index
HF: High-Frequency Power
LF: Low-Frequency Power
HF/LF: Ratio of High-Frequency Power to Low-Frequency Power
LVAD: Left ventricular assist device
Hb: Hemoglobin
HDL: High-density lipoprotein
LDL: Low-density lipoproteins
DNA: Deoxyribonucleic acid
BMI: Body mass index
SBP: Systolic blood pressure
DBP: Diastolic blood pressure
SNCA: Synuclein alpha
PPARG: Peroxisome proliferator-activated receptor gamma
XRCC: X-ray repair cross-complementing 1
FLACC 1: flagellum associated containing coiled-coil domains 1
FTO: FTO alpha-ketoglutarate dependent dioxygenase
CAN- group: The group without CAN
CAN + group: The group with CAN
OR: Odds ratio
95%CI: 95% confidence interval
